# Digital twins for health: a scoping review

**DOI:** 10.1038/s41746-024-01073-0

**Published:** 2024-03-22

**Authors:** Evangelia Katsoulakis, Qi Wang, Huanmei Wu, Leili Shahriyari, Richard Fletcher, Jinwei Liu, Luke Achenie, Hongfang Liu, Pamela Jackson, Ying Xiao, Tanveer Syeda-Mahmood, Richard Tuli, Jun Deng

**Affiliations:** 1 VA Informatics and Computing Infrastructure, Salt Lake City, UT 84148 USA; 2 Department of Radiation Oncology, University of South Florida, Tampa, FL 33606 USA; 3 Department of Mathematics, University of South Carolina, Columbia, SC 29208 USA; 4 Department of Health Services Administration and Policy, Temple University, Philadelphia, PA 19122 USA; 5 Department of Mathematics and Statistics, University of Massachusetts Amherst, Amherst, MA 01003 USA; 6 Department of Mechanical Engineering, Massachusetts Institute of Technology, Cambridge, MA 02139 USA; 7 Department of Psychiatry, Massachusetts General Hospital, Boston, MA 02139 USA; 8 Department of Computer and Information Sciences, Florida A&M University, Tallahassee, FL 32307 USA; 9 Department of Chemical Engineering, Virginia Polytechnic Institute and State University, Blacksburg, VA 24060 USA; 10 McWilliams School of Biomedical Informatics, University of Texas Health Science Center at Houston, Houston, TX 77030 USA; 11 Precision Neurotherapeutics Innovation Program & Department of Neurosurgery, Mayo Clinic, Phoenix, AZ 85003 USA; 12 Department of Radiation Oncology, University of Pennsylvania, Philadelphia, PA 19104 USA; 13 IBM Almaden Research Center, San Jose, CA 95120 USA; 14 Department of Therapeutic Radiology, Yale University, New Haven, CT 06510 USA

**Keywords:** Drug development, Clinical trial design

## Abstract

The use of digital twins (DTs) has proliferated across various fields and industries, with a recent surge in the healthcare sector. The concept of digital twin for health (DT4H) holds great promise to revolutionize the entire healthcare system, including management and delivery, disease treatment and prevention, and health well-being maintenance, ultimately improving human life. The rapid growth of big data and continuous advancement in data science (DS) and artificial intelligence (AI) have the potential to significantly expedite DT research and development by providing scientific expertise, essential data, and robust cybertechnology infrastructure. Although various DT initiatives have been underway in the industry, government, and military, DT4H is still in its early stages. This paper presents an overview of the current applications of DTs in healthcare, examines consortium research centers and their limitations, and surveys the current landscape of emerging research and development opportunities in healthcare. We envision the emergence of a collaborative global effort among stakeholders to enhance healthcare and improve the quality of life for millions of individuals worldwide through pioneering research and development in the realm of DT technology.

## Background

In the study of complex dynamical systems, a cost-effective way of studying the implication of different design choices and options is through simulation of the entities of interest through virtual models. The virtual models, called the digital twins, are a virtual representation of objects spanning their development and progression lifecycles to help in the overall decision making. In industrial manufacturing, digital twins are used throughout the product lifecycle to simulate, predict, and optimize the product and production system before investing in physical prototypes and assets^1^. A digital twin (DT) can be more than just a digital replica or a virtual model of a physical system, it can be a sophisticated representation designed to faithfully mirror the real-world system in real-time, analyze its behavior, and provide predictive insights using advanced simulation, machine learning and reasoning to help decision making. The analytical and predictive capability of a DT makes it distinct from a dummy replica of the physical system^2^.

The DT concept was first adopted by the NASA space program in the 1960s to simulate a spacecraft and be able to debug flight issues in real-time as they arose. This concept was successfully utilized during the Apollo 13 mission when the spacecraft suffered a malfunction and the NASA team had to simulate conditions aboard Apollo 13 to bring back the spacecraft and astronauts safely to Earth^3^. The actual term “digital twin” (DT) was then coined in 2005 by Michael Grieves in product lifecycle management^4^. Around 2010, NASA and John Vickers utilized the DT as a virtual model of a physical system^5,6^. In the original description, a DT is characterized by three components: physical, virtual, and a connection, where the virtual system is mapped to the physical system by exchanging information through a real-time data connection.

Multiple types of digital twins (DTs) have been proposed in the literature, highlighting the evolution of the DT concept. Initially, the DT was described as a static twin, representing a digital replica of a physical system. However, as the concept advanced, new iterations emerged, including the “mirror twin”, the “shadow twin”, and more recently, the “intelligent twin”^1,2^. A static twin model has only static properties while a functional twin (also known as a mirror twin) is a static twin with dynamic behavior capabilities. Some examples of mirror twins are demonstrated in Sections on DTs for Surgical Planning and DTs for In-Silico Clinical Trial Design and Intelligent Randomized Control Trials. The self-adaptive twin (also known as the shadow twin) is a functional twin with the capacity to acquire real-time data and update the model, requiring a digital thread keeping track of evolution and communication with the real-world object, system, or organism. Sections on DTs for Medical Device Design, DTs for Biomarker and Drug Discovery, and DTs in Biomanufacturing list some examples of shadow twins. The most evolved version of the DT is the intelligent DT which is a self-adaptive twin with a degree of artificial intelligence that has autonomy with learning, reasoning, knowledge, and acting capabilities, and can communicate with other twins (also known as extended DTs, cognitive DTs, or physical avatars)^7^. This DT needs information exchange between the physical world and the DT in both directions dynamically. The continuous connection and exchange of information between the physical and virtual worlds enable the optimization of simulation and machine learning algorithms to analyze and make predictions on the future state of the real system, optimizing the system and accelerating decision-making^8^. Some applications of intelligent DTs in healthcare are discussed in Sections DTs for Hospital Management Design and Care Coordination, DTs for Personalized Medicine, and DTs for Wellness.

Recently, the refinement and convergence of technologies such as Generative Artificial Intelligence, Cognitive Computing (CC), Internet of Things (IoT) and sensors, have enabled the practical applications of DTs in diverse sectors including aerospace, automobile, energy management, urban planning, construction, environmental management, climate modeling, and healthcare industry.

In the field of medicine, the DT concept has also begun to emerge, particularly in the fields of precision medicine, cancer care, individualized training, and personal well-being^2,8^. DT applications for healthcare include hospital management, facility design, workflow development, decision making, as well as individualized therapy and personalized patient care. DTs may be adapted as a well-being DT or in a state of illness for personalized diagnosis, treatment planning, care, and survivorship. We envision that the DT concept can bring many opportunities to healthcare including in silico clinical trial design, medical device design, drug discovery, care coordination, treatment design, and surgical planning, etc. Each person is unique, and it is important to collect deep digital phenotypes when developing patient-specific DTs. In this review, we appraise the utility of the digital twin across a wide spectrum of sectors of the healthcare industry as well as emerging real-world use cases, which are often enabled through consortium approaches that allow stakeholders to leverage combined resources and technologies.

### Scope of the review

#### Definition of DT for healthcare

**Fig. 1:**
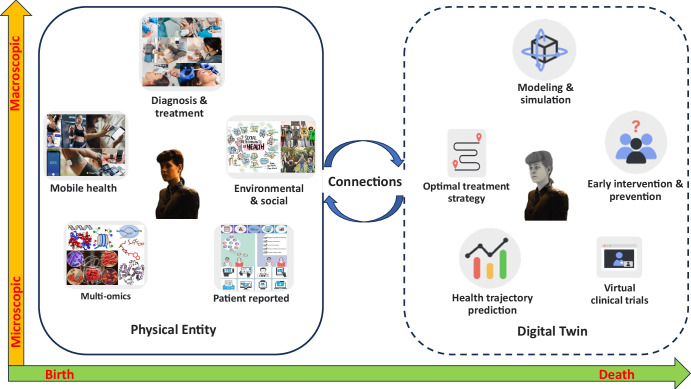
Digital twin for health (DT4H) envisioned.

In combination of our perspectives with many other definitions and perceptions in the literature^2,7–11^, we define a digital twin for healthcare as a virtual representation of a person which allows dynamic simulation of potential treatment strategy, monitoring and prediction of health trajectory, and early intervention and prevention, based on multi-scale modeling of multi-modal data such as clinical, genetic, molecular, environmental, and social factors etc. As shown in Fig. 1, the main components of a DT consist of a physical entity, a virtual replica, and a connection between the two to enable bi-directional real-time impact on each other. The ever-evolving interactions between the physical entity and the digital twin could traverse from microscopic to macroscopic scale and last from birth to death for individuals. A DT should be **i**ndividualized, **i**nterconnected, **i**nteractive, **i**nformative, and **i**mpactful (5Is). In the context of a patient-specific DT, on one hand, the simulation, prediction, and analysis from DT can be used to help the patient with better treatment outcomes and less adverse effects. On the other hand, the real-world data from the patient can be used to benchmark, validate, and improve the DT modeling.

Recently, computational modeling and AI/ML algorithms have been widely used in disease models, target identification, in silico trial simulations, virtual or synthetic patients, virtual coaches, and personalized medicine. While they form an important approach toward virtual replica of a physical entity, neither computational modeling nor AI/ML algorithm is the totality of DT. To classify a system (a person, an organ, a disease, or a drug) as a DT, it must have the three components (a physical entity, a virtual replica, and a connection between the two) and be individualized, interconnected, interactive, informative, and impactful.

#### AI and ML modeling

The nuances and distinctions among various virtual models are paramount, particularly when considering aspects such as data sources, applications, interactions, and visualization methods. For instance, DTs that simulate specific organs, like the living heart model, are mainly developed using detailed imaging data. This contrasts sharply with disease models aimed at supporting precision medicine, which are underpinned by a rich mix of molecular profiling data and clinical information.

The application of these models is as diverse as their data sources. Organ-specific digital twins, such as those replicating heart function, are crucial in predicting how mechanical medical devices (like pacemakers and stents) will perform. On the other hand, disease models provide invaluable insights into the effectiveness of pharmaceutical interventions, especially in how drugs interact with complex biological processes. Beyond these differences, the computational analytics employed in each model type also vary significantly. This variance not only reflects the different needs and objectives of each model but also underscores the specialized nature of the analytics involved. Understanding these differences is crucial for leveraging the full potential of digital twins in improving healthcare outcomes and advancing medicine.

#### Literature review: search strategy and data collection

To systematically explore the landscape of research on digital twins in the context of health, we conducted a comprehensive literature search using PubMed. Our search strategy was twofold: (1) Search for “Digital Twin” and Health (2016–2023): The first part of our search was aimed at identifying articles that explicitly discussed digital twins in relation to healthcare applications. We used keywords “digital twin” in combination with “health” to filter relevant articles and the timeframe was restricted to the years of 2016 to 2023.

**Fig. 2:**
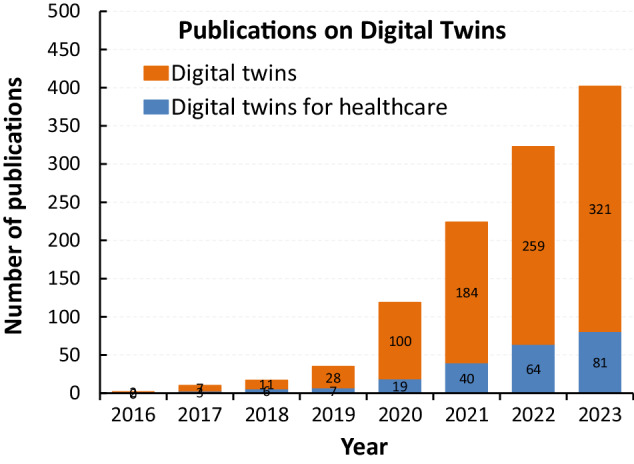
The trend of publications on digital twins from 2016 to the end of November 2023.

(2) Search for “Digital Twin” by year of publication (2016–2023): Concurrently, we conducted a year-wise search for articles using the keyword “digital twin” without health. This was intended to capture the overall growth of DT technology in literature, irrespective of the domain. By comparing the findings of both searches, we aimed to contextualize the trajectory of DT research within health sector as shown in Fig. 2.

Out of these 220 papers of DT in healthcare, we have selected 85 representative references which form the basis for the Sections 3 to 5.

#### Publication trend on DT for healthcare

The field of digital twins for health applications has experienced a significant surge in publications in recent years. Researchers and experts have recognized the potential of digital twins to revolutionize healthcare delivery, patient care, and medical research. There has been a growing interest in exploring and harnessing the capabilities of digital twins in various health-related domains as shown in Fig. 2. In addition to the increasing number of publications each year, the ratio of the number of publications on digital twins for healthcare to the number of publications on digital twins in general has been steadily increasing over the years, which further suggests that digital twins for health have increasingly attracted the attention of the researchers around the world.

#### Key technologies used in DT for healthcare

**Fig. 3:**
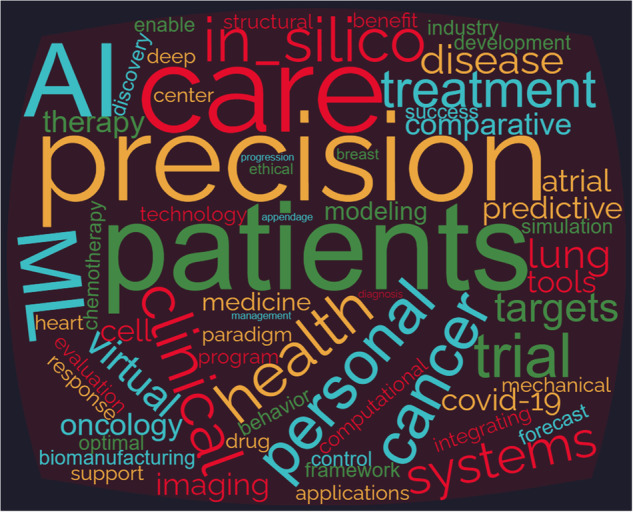
The word cloud generated from the titles of the papers reviewed in this study.

The word cloud generated from the titles of the publications reviewed in this paper is summarized in Fig. 3. The visualization displays words that appear at least twice in the titles. The size of each word represents its frequency in the titles. Previous studies on DT have primarily concentrated on patient care, with a specific focus on cancer, particularly lung and breast cancer, as well as cardiovascular diseases. Additionally, these studies have explored clinical trials and various other diseases, all aimed at advancing personalized and precision treatment approaches in healthcare. The tools and technologies utilized in these investigations are primarily based on Machine Learning (ML) and Artificial Intelligence (AI), specifically for modeling, simulations, and creating virtual representations. Moreover, prior research has also delved into establishing the paradigm and addressing ethical considerations related to the application of DT in healthcare.

Furthermore, the increase in digital twin publications for health reflects the interdisciplinary nature of the field. It encompasses collaborations between experts in medicine, engineering, computer science, data analytics, and other disciplines. By combining expertise from multiple domains, researchers aim to advance the development and implementation of digital twins to address critical healthcare challenges.

### Different types of DTs for healthcare

In terms of DTs for healthcare, different DT types may be conceived. As shown in Table 1, there are DT types of one body system, or body organ (i.e., lung or heart) or body function, or finer body component levels (cellular, subcellular) or of the entire human body^12–14^. Similarly, DTs can be created for a specific disease or disorder. Composite DTs may integrate two or more of the different types of DTs. The DT’s fidelity largely depends on incorporating real-time and real-world information (e.g., clinical sensors’ real-time updates and encapsulated autonomy of the DT). A DT instance (or realization) describes the physical twin during its entire lifespan. A DT aggregate is the aggregation of some of the DT instances and other DT aggregates. A further illustration of the DT is the DT bank, which is an organized repository of instances as well as aggregates. DT banks may be useful for clinical trial matching and drug development. The DT thread, on the other hand, is a temporal data pipeline from birth to death, which tracks the relations of data elements over time^15^.

**Table 1:** Various types of DTs in healthcare

Physical Entity	Entity	Mechanism	Endpoint
Lung	Lexma^12^	Runs simulations of blood and oxygen flow	Predict ventilation requirements
Heart	Dassault^13^, Medtronic, Boston Scientific, FDA;	Simulates the structure and physiologic function of the heart	Customization and optimization of cardiac devices
Heart	Siemens Healthineers^23^	Simulates the structure and physiologic function of the heart	Cardiac resynchronization
Heart	Heart Navigator^40^	simulated TAVR implantations with different aortic prosthesis	Surgical planning
Spine	Ahmadian et al.^42^	Predict Vertebral Fracture after Stereotactic Body Radiotherapy	Optimal radiation plan to minimize treatment side effects
Alzheimer’s disease	Unlearnai^14^	Predicting the individual outcome in neurological diseases	DT of controls of clinical trial and ultimately clinical interventions
Breast lesions	VICTRE trial^51^	Image based virtual patients comparing digital mammography to tomosynthesis	Determine which imaging tool is better at detecting breast lesions
Oropharyngeal cancer	Tardini et al.^61^	Optimal treatment selection	Determine optimal treatment plan for oropharyngeal cancer
Type 2 Diabetes	Cleveland Clinic Twin Health NCT05181449^88^	Disease reversal in type 2 diabetes	Randomized control trial examining twin precision treatment vs. standard of care
Mental health	MindBank AI^65^, IBM^66^, Babylon^67^, DigiTwin^68^		Wellness
Pharma Lab	Atos, Siemens, GSK^23,36^		Optimize drug manufacturing
Biomanufacturing	Teva and AG Pharmaceuticals^37^	Adjust input conditions Key and Critical Process Parameters	Predictive biomanufacturing
Drug Discovery	Takeda^34^		Drug Discovery
Hospitals	GE Care command^24^; Siemens Healthineers^23^		Earlier response times for critical patients, supply chain management, workflow

### Various applications of DTs for healthcare

**Fig. 4:**
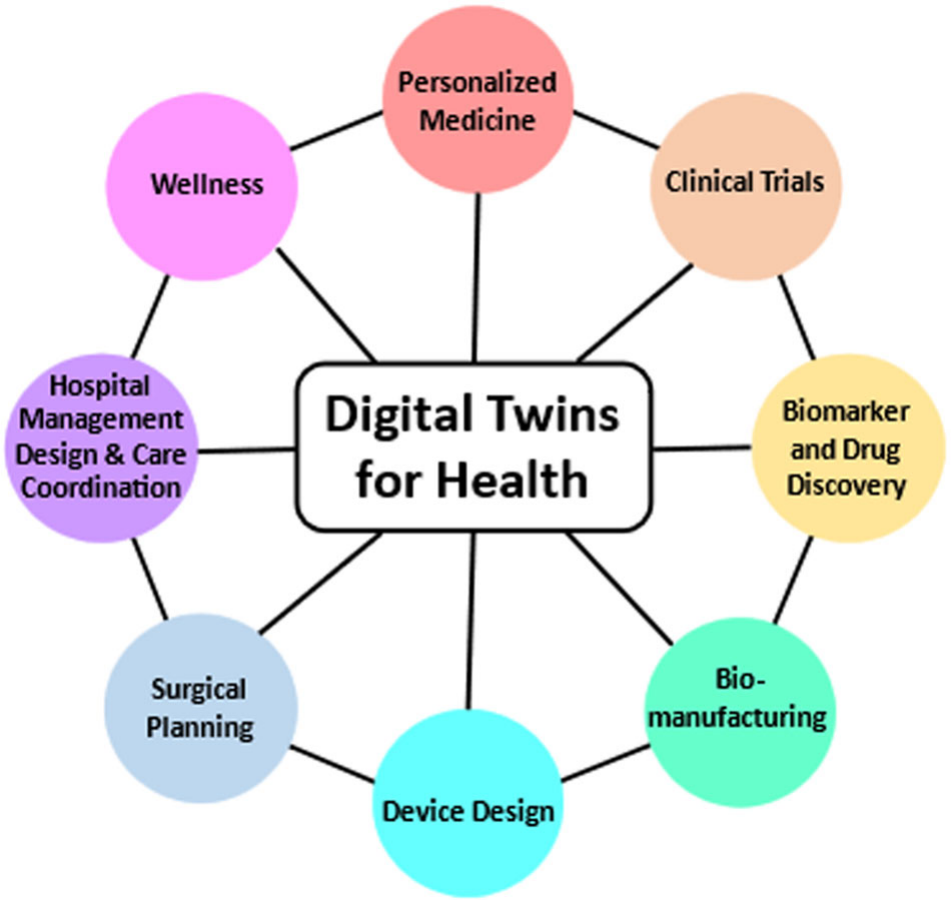
Main applications of digital twins for health. (1) Hospital Management Design & Care Coordination; (2) DeviceDesign; (3) Biomarker and Drug Discovery; (4) Bio-manufacturing; (5) Surgical Planning; (6) Clinical Trials; (7)Personalized Medicine; and (8) Wellness.

In reviewing applications of DT4H up to date, we roughly pigeonhole them into 8 different categories based on the purpose and the contents of the application. Figure 4 summarizes the 8 main applications of DT4H.

### DTs for hospital management design and care coordination

DTs have been used in healthcare to build a digital representation of healthcare data (electronic medical data), hospital environments, physical hospitals, human physiology, operational staff, and lab results^7,16^. The applications are wide-ranging, encompassing the efficiency of resource utilization to minimize resource shortages, manage hospital workflow, revolutionize clinical care processes, and directly enhance patient care^17^. DTs in the context of healthcare management have been classified into three types: (1) processes or production (for efficient design of new products), (2) product design (for use in manufacturing and production planning), and (3) system performance (for the capture, aggregation, and analysis of operational data).

#### Processes or production

DTs of the processes in operation controls are being developed to support the process of severe trauma management^8^, the intensive care unit station^18^, the supply chain process, and cybersecurity^17^. In trauma management, the DT state starts before the patient’s arrival in the unit and the collection and information receipt occurs directly from the accident site, allowing for earlier response times which are critical when time is of the essence. In the ICU station DT, anomalies may be detected earlier and intervened at an earlier stage.

#### Product design

DTs are also being incorporated into information management and the promotion of well-being. DTs are embedded in wearable devices that gather personal information. Liu et al. propose a cloud-based framework for CloudDTH which uses personal data from digitally twinned wearable medical devices aimed at real-time supervision with crisis warning to ultimately support the self-management of the elderly^19^. Wickramasingh proposed a DT framework for dementia care^19,20^.

The DTCoach is a DT mentor that can be used on smartphones and provides person-centered digital coaching during the COVID-19 pandemic^17,21^. DTs have been used to provide alternatives to telehealth such as transparent remote doctor visits and robot-assisted and AI-assisted telerehabilitation with bidirectional presence control^22^.

#### System performance

Siemens Healthineers and the Medical University of South Carolina have partnered to improve DT applications by simulating changes in workflows and medical equipment on hospitals’ efficiency^23^. GE health systems have already established DT of the hospital (Care Command) to optimize workflow and care coordination. Specifically, during the COVID-19 pandemic, which was akin to a digital stress test and a crisis, predictive models in Oregon showed a high likelihood of exceeding critical care resources such as ventilators and critical care beds as well as access to extracorporeal life support (ECLS) and GE Healthcare command center real-time data-tracking tool concept was utilized across the state^24^. The command center technology was used to track bed and ventilator capacity data across all hospitals in the entire state which maximized the utilization of critical resources and mitigated the risk of decision-making in silos without examining statewide resources. Generation 2 of the command center technology supports early identification of real-time management of patients at risk of harm and deterioration^25^. Visual displays of real-time data are presented from which early actionable interventions can be implemented to manage patients who are at risk of harm and deterioration and to eliminate any events or medical errors that should never happen^17^.

### DTs for medical device design

The concept of virtually customizable DT models for organs has gained attention and advancement. An example of such an initiative is the collaboration between Dassault Systèmes and the US Food and Drug Administration (FDA) in a project called, SIMULIA Living Heart^13,26^. The Living Heart project was launched in 2014 and the aim was to crowdsource a virtual twin of the heart. This is now a validated DT model of the human heart and examines in silico organ drug interactions. With additional partnerships, including Philips and Siemens Healthineers, a DT twin of the heart is also being used to develop and refine cardiac device design at a faster pace. In Europe, FEops aims to change the course of structural heart disease management by combining a digital twin of the heart with AI-enabled anatomical analysis in all aspects of patient care from patient selection, procedure planning, periprocedural guidance, to follow-up^27^. In the EU, UK, Canada, and Australia, FEops HEARTguide is available for AI-enabled anatomical analysis and computer simulation in Transcatheter aortic valve implantation (TAVI) and Left Atrial Appendage Occlusion (LAAO) and is FDA cleared in the US^28^.

Indeed, the development of the DT for organs has extended to the lungs, particularly in the context of predicting ventilation requirements and optimizing patient outcome^29^. The DT of the lungs expanded during the Covid pandemic and as part of Project BreathEasy, Onscale developed the DT of the lungs of COVID-19 patients to improve patient outcomes and optimize the use of limited ventilator resources, particularly in areas heavily affected by the outbreak^30^. The Living Brain project provides a tracking progression of neurodegenerative disease^31^. The long-term goal is to create a DT of the entire human body. As technology continues to advance, the potential of DT models for organs is expected to expand further, leading to more precise and personalized medical interventions, improved patient outcomes, and enhanced resource management in the healthcare sector.

### DTs for biomarker and drug discovery

Conventional drug discovery is risky, expensive, and time-consuming, involving target identification, and validation followed by preclinical and clinical trials. The estimated cost of bringing a new drug to market is about $2.6 billion US dollars and the time to market is about 10 years. Moreover, the attrition rate of drug targets has been reported to be as much as 96%^32^. Various computer-aided drug discovery (CADD) approaches which are selected based on the availability of target protein structural information such as structure-based drug discovery (SBDD) and ligand-based drug discovery (LBDD) have been integrated with machine learning techniques to optimize CADD and high-throughput screening of absorption, distribution, metabolism and excretion, and toxicity (ADME-Tox) properties in drug discovery and development^33^.

DT modeling shortens the pharmaceutical processes and makes realistic input–output predictions for biochemical reactions^34^. Numerous drugs identified using in silico techniques have reached the market for a variety of diseases including human immunodeficiency virus (HIV)- 1 inhibiting drugs (atazanavir, saquinavir, indinavir, and ritonavir, anti-cancer agents (raitrexed), and antibiotics (norfloxacin)^32^. DTs are speeding up this process by identifying drug targets that are more likely to succeed^35^. Atos, a leading name in digital transformation, and Siemens have collaborated with the pharmaceutical companies (e.g., GSK) to optimize the drug manufacturing process through the physical DT model of the pharma laboratory process^36^. Takeda Pharmaceuticals, a Japanese multinational pharmaceutical company, has switched to DT technology in production to launch transformative therapies globally. Deploying DTs to mimic various potential biochemical reactions in drug design could significantly reduce the drug development circle to benefit mankind.

### DTs in biomanufacturing

Biomanufacturing relies on naturally occurring processes and reactions that permit the consistent production or output of biological products at a commercial scale. Specifically, these products may include medicine and vaccine production, antibacterials, on-demand molecule production, and on-demand tissues and organs.

In silico Biotechnology AG and Teva Pharmaceuticals have also partnered to apply in silico technology for predictive biomanufacturing to create and implement more efficient production processes of biopharmaceutical therapies. In the virtual DT system, multi-scale mechanistic and data-driven models are combined to mathematically relate adjustable input conditions (e.g., key process parameters (KPPs) and critical process parameters (CPPs) such as media compositions and bioreactor conditions PH, temperature, and dissolved oxygen) with output responses (e.g., key performance indicators (KPIs) such as titer and yield and critical quality attributes (CQAs))^37^. It is currently tested to be successful and is supported by the IoT, AI, and many other advanced technologies^38^.

Furthermore, these applications are not limited to the biomolecule production and extend to the synthesis of chemical molecules. For instance, the 4th Industrial Revolution has transformative effects on production processes^39^. In the pharmaceutical sector, its implementation simplifies complex drug manufacturing through technologies like AI, robotics, and IoT. One of the main goals of Industry 4.0 is to design innovative, tailor-made products that cater to the ever-evolving demands of customers, while maintaining economic and operational efficiency, reducing the need for human labor in challenging pharmaceutical processes.

### DTs for surgical planning

Surgery is a complex process with many opportunities for error which can result in adverse or fatal outcomes for patients. The DT of anatomical structures can help surgeons simulate surgical approaches before the actual procedure^26^. The DT of heart models (HeartNavigator) has been developed for utilization before complex cardiac procedures, such as transcatheter aortic valve replacement (TAVR) surgical procedures. The computer-simulated “virtual TAVR” to guide TAVR has been reported. Specifically, simulated TAVR implantations with a different aortic prosthesis at their recommended implant depth were explored^40^.

The utilization of DTs in orthopedics is revolutionizing surgical procedures by helping surgeons’ study medical implants before surgery. In orthopedics, DTs are being developed to assist surgeons to choose the optimal stabilization method and optimal postoperative treatment based on individual patient characteristics^41^. DTs of trabecular bone using Deep Convolutional Generative Adversarial Networks are being used to simulate vertebroplasty operations and their impact on vertebral fracture responses^42^. In a cancer patient, a DT is being utilized to predict the risk of vertebral fracture in metastatic spine cancer patients after stereotactic body radiotherapy (SBRT) to assist in informed decision-making and design of better treatment strategies to benefit these patients^43–46^.

### DTs for in-silico clinical trial design and intelligent randomized control trials

Clinical trials are costly and time-consuming. A developing strategy is an in-silico trial (IST), which is a clinical trial that is conducted digitally through simulation and modeling. Less than 10% of adult cancer patients participate in clinical trials and oncology has one of the lowest rates of success in bringing a drug to market approval, with likelihood at about 5.1%^47,48^. While structural barriers exist, a large portion of the costs of clinical trials are related to patient recruitment which is especially challenging in rare diseases where the standard of care therapies may not exist or where patients do not want to risk randomization to the standard of care or placebo. All these factors contribute to underpowered clinical trials or clinical trials that fail. ISTs can support better-powered trials, simulate both control and efficacy arms, and ultimately optimize patient recruitment and drug protocols^49^. Digital twins are different from external control arms that are incorporated into clinical trials. External controls are selected from external data sources such as historical clinical trials, and real-world data such as electronic health records or registries. Digital twins add predicted outcomes of individual patients’ model-based estimates of what the individual outcome would have been in the control group. Companies such as Unlearn.AI are designing trials in diseases where there is a significant amount of data, mainly neurological diseases such as Alzheimer’s, Parkinson’s, and multiple sclerosis^14^. While DTs are being developed, there has been increased confidence in in-silico tools, and in-silico trials have been recognized in both the development and regulatory approval of medical devices, as control arms in clinical trials.

There are several in silico simulations for predicting the response to treatments and suggesting the optimal drug dosages for each patient based on their disease’s characteristics^50^. Moreover, the use of in-silico trials in evaluating medical imaging technologies has been demonstrated by the VICTRE (the Virtual Imaging Clinical Trial for Regulatory Evaluation) study^51^. By utilizing computer-simulated images of virtual patients, the study compared the performance of digital mammography and digital breast tomosynthesis for detecting breast lesions. The in-silico trial involved a large cohort of 2986 synthetic image-based virtual patients, and the results were compared to those of a clinical trial where 400 women received both imaging methods. The findings of the in-silico trial correlated well with the clinical trial, indicating that digital breast tomosynthesis was more effective than standard-of-care digital mammography in detecting breast lesions. This example showcases the potential of in-silico trials as a complementary tool to traditional clinical trials. In-silico trials can provide valuable insights and predictions regarding the performance and efficacy of medical devices or interventions, allowing for more efficient and cost-effective evaluations. The FDA advocates for in silico modeling and simulation and acknowledges the potential of computational modeling to enhance the regulatory assessment process. They have also issued guidance on the use of computational modeling in medical device development^52^.

In-silico clinical trials are first being studied in the form of synthetic control arms and ultimately for predicting clinical intervention arms. Both the FDA and European Medicines Agency (EMA) have taken initiatives to support the integration of in-silico approaches to control arms. A synthetic control arm of 68 patients was used to expand coverage across 20 European countries of targeted therapy for non-small cell lung cancer, alectinib^53^. Due to synthetic controls, Palbociclib, a kinase inhibitor had expanded indications for men with HR-positive Her2-negative advanced or metastatic breast cancer, and blinatumomab used to treat a rare form of acute lymphoblastic leukemia, received accelerated approval^54^.

Synthetic controls have been utilized in cancer, hepatitis C, as well as rheumatoid arthritis^55^. The current landscape of in-silico trials is evolving and incorporating multimodal clinical, genomic, radiomic, and socio-economic data, enabling the more robust design of synthetic controls and the prediction of clinical intervention outcomes through artificial intelligence. ISTs sustained by AI undergo iterative processes where new patient data is incorporated with each simulation and learns from prior simulations to enhance predictions. The crux of IST design is the ability to recreate human physiology and pathology based on genetics and environment. Recently, in-silico modeling has been used to predict responses and identify patients that may benefit from immunotherapies to improve clinical trial design^49,56^. A DT designed for clinical trials would be augmented with more onboard analytical and intelligent functions to make adaptive decisions and suggestions to physicians to mimic intelligent human responses.

### DTs for personalized medicine

In the field of oncology, precision oncology and the increasing availability of Comprehensive Genomic Profiling (GCP) have changed the landscape of cancer therapy by providing information about biomarkers that are targetable by precision tumor therapies. For physicians reviewing CGP results, identifying a mutation-bearing level 1 therapeutic level of evidence (LOE), defined as a gene variant recognized by the FDA for predicting the response to an FDA-approved drug for a patient’s specific indication, is most relevant^57^. Unfortunately, rates of mutations with level 1 LOE occur in a minority of patients, biomarkers with lower LOE for TAs are more frequently observed and a small portion of patients benefit from precision medicine. Deep phenotyping has been defined as the precise and comprehensive analysis of phenotypic abnormalities, in which the phenotype is observed, and includes genetic, clinical, eHealth records, and biomedical data which will lead to personalized oncology^58,59^.

To fully achieve personalized care for each patient, features from each individual (digitosome or digital phenotype) which is all real-world data generated digitally online, via smartphone or other connected devices must be integrated into a deep digital phenotype^10,58^. Digital data may provide additional information regarding individuals’ lifestyles, psychological states, socio demographics, and the environment of an individual which may affect whether a therapeutic strategy will succeed or fail.

In the field of clinical oncology, digital twin blueprints and examples of practical development of digital twin framework focusing on image-guided mechanisms and adaptive radiotherapy for high-grade gliomas have been proposed^60^. In a recent study, patient-physician digital twin dyads were developed to simulate therapy outcomes and determine the optimal treatment selection for oropharyngeal carcinoma, specifically whether patients would benefit from sequential versus concurrent chemotherapy and radiation with high accuracy^61^. In another study with triple-negative breast cancer (TNBC), quantitative MRI and biologically based mathematical modeling were used to successfully predict responses to the neoadjuvant chemotherapy, suggesting digital twins could predict and optimize cancer therapy^62^. More recently, DTs of patients with non-small cell lung cancer were created to predict the optimal salvage therapy after disease progression on pembrolizumab. Over 25,000 lesions measurements, from >500 patients were simulated in terms of response to pembrolizumab, chemotherapy, and Progressive Disease on pembrolizumab followed by either pembrolizumab beyond progression or salvage chemotherapy^63^. Switching all progressors to salvage chemotherapy was suboptimal and pembrolizumab was found to be beneficial in patients with progressive disease in the nontarget initial lesions^63^. Many groups are exploring approaches for predictive cancer patient DTs through team science and collaborative efforts^64^.

### DTs for wellness

While some DTs target a specific organ or disease, others are providing general products which may improve personalized health^7^. Some DTs focus on self-reflection and redefining quality mental health with loop feedback to improve well-being such as MindBank Ai^7,65^. Babylon is capturing health data from fitness devices and wearables and then transforming them into DTs that then support engagement between doctors and patients. In addition, IBM^66^, Babylon^67^, and DigiTwin^68^ use DT technology to deliver personalized healthcare services encouraging wellness.

The use of digital phenotyping methods, described previously, is now revolutionizing the fields of psychiatry and behavior medicine which enable daily monitoring of psychological states and health behaviors to support individual wellness^69^. Academic research platforms, such as Beiwe^70^ and Mindlamp^71^ are now being used to develop personalized models that enable behavioral and psychological interventions that can be delivered in real-time^72^. Although digital twin models of the human brain are not yet possible, it is hoped that future digital twin technology will greatly improve the current practice of clinical psychopharmacology.

### DT healthcare research centers or consortiums

DT-based healthcare research centers have been developed to improve and expand the landscape of DT technology and applications for the goal of ultimately improving patient care and personalizing wellness, disease prevention strategies, diagnosis, prognosis, and treatment. Digital twin consortia bring together academia, industry, and government and will play an important role in the standardization of digital twin methods and interoperability protocols. Examples of DT centers include the Swedish Digital Twin Consortium (SDTC), Empa research center, Human Digital Twin OnePlanet research center, DIGIPREDICT consortium, PRIMAGE, MAI DigiTwin, The Digital Twin Consortium, and The Digital Twins for Health Consortium (DT4H.org), which are summarized in Table 2. Each center has its focus and strengths.

**Table 2:** DT Healthcare Research Centers around the world

Research Center	Community	Emphasis	Aims
Swedish Digital Twin Center SDTC	Swedish multi-disciplinary healthcare and industry	single-cell RNA seq-based framework	Precision medicine, biomarkers for personalized treatment, new drug candidates, time-dependent personalized prescriptions of drug combinations
Empa Research Center	Swedish multi-disciplinary healthcare and industry	Opiates and twinning of transdermal drug delivery	Optimize drug dosage for persons afflicted by chronic pain
Human DT One Planet Research Center	Netherlands	Gut microbiome, nutrition, behavior, and identification of lifestyle-related diseases	Optimize diet and lifestyle for disease prevention
DIGIPREDICT	A consortium of five European countries	Cardiovascular, infectious diseases, & COVID-19	Early intervention to halt disease progression in infectious and cardiovascular disease
PRIMAGE **PR**edictive **I**n-Silico **M**ultiscale **A**nalytics to support cancer personalized dia**G**nosis and prognosis, **E**mpowered by imaging biomarkers	Multi-disciplinary European	Imaging biomarkers and childhood solid malignancies, including neuroblastoma and diffuse pontine glioma	Imaging biomarkers to support clinical decision-making including treatment management to optimize patient outcomes
MAI Medical Augmented Intelligence DigiTwin	United States and Taiwan	Converts 2D images to 3D virtual images for patient education	Medical learning through DT for both patients and practitioners
The Digital Twin Consortium	Multi-disciplinary from industry, academia, and government. Based in the US and has a global membership	Open to any entity with an interest in DT	Multipurpose DT software development across many sectors
The Digital Twin for Health Consortium^78^	Multidisciplinary DT enthusiasts interested in developing the digital twin infrastructure for healthcare	Develop DTs for better health in collaboration with all the stakeholders in the healthcare spectrum	On-going projects on digital twins for lung cancer, sepsis, mental health diseases, leukemia, and cardiovascular diseases

The Swedish Digital Twin Consortium (SDTC) is a national initiative aimed at developing a strategy for personalized medicine using single-cell RNA sequencing (scRNA-seq). Medications are ineffective in 40–70% of common diseases. An important cause of this high rate of failure is suggested by genome-wide association studies (GWAS), which identify increasing numbers of genetic variants that may affect cell types in the same disease. Single-cell expression studies have revealed altered gene expressions of thousands of genes^73^. The SDTC strategy is based on (i) constructing unlimited copies of network models of all molecular, phenotypic, and environmental factors relevant to disease mechanisms in individual patients (i.e., digital twins); (ii) computationally treating those digital twins with thousands of drugs to identify the best-performing drug; and (iii) treating the patients with this drug^73^. Another Swedish-based DT platform is the Empa research center which uses customized DT (utilizing age and lifestyle) to optimize the dose of pain medications. Patients feed data into the DT and improve the accuracy of the DT by reporting on the effectiveness of the customized dose^74^.

The Human Digital Twin, OnePlanet Research Center, in collaboration with nutritionists and behavioral health experts, is developing an AI-guided, digital platform for the continuous collection, integration, and analysis of behavioral, health, and nutrition data. Human Digital Twin is a self-learning system that aims at predicting the optimal diet and lifestyle interventions for an individual^75^. The DIGIPREDICT DT consortium proposes a DT that predicts disease progression and the need for early intervention in infectious and cardiovascular diseases^76^.

Other initiatives focus on specific populations, such as PRIMAGE, and PRedictive In-Silico Multiscale Analytics to support cancer personalized diaGnosis and prognosis, Empowered by imaging biomarkers. PRIMAGE is one of the largest European research initiatives involving artificial intelligence in childhood malignancies. The project is constructed as an observational in-silico study involving anonymized datasets (imaging, clinical, molecular, and genetics) for the training and validation of machine learning and multiscale algorithms^77^.

Medical Augmented Intelligence (MAI) has a DigiTwin platform that converts 2D patient medical images (MRI and CT scans) into 3D virtual images in less than 30 seconds^68^. This technology enables clinicians to energize and involve patients with their DT for shared decision-making through improved education. There are also groups such as the Digital Twin Consortium, which caters to all sectors and industries ranging from Aerospace to Agriculture. Ultimately, it is anticipated that a multipurpose DT software development tool kit will become available^15^.

The recently formed Digital Twins for Health Consortium (DT4H.org) is a self-assembled organization, comprising multidisciplinary DT enthusiasts who are interested in developing the digital twin infrastructure for health to revolutionize the healthcare paradigm^78^. Members of the consortium have ongoing projects on digital twins for lung cancer, sepsis, mental health diseases, diabetes, leukemia, and cardiovascular diseases, and published jointly on some latest results^64^.

### Challenges and recommendations of DTs for healthcare

While this is not a methodological review of DT technologies, we however, point out the following known challenges impeding the developments in this field below:

### Challenges

#### Data acquisition and integration

One of the main challenges in the clinical translation and development of a DT for health is the acquisition of accurate data with real-time synchronization and multimodal fusion. The integration of physiological, biological, and chemical models into DT simulations that capture the underlying pathways and disease processes is becoming an emerging trend that will enable a much higher degree of customization and adaptability. Acquiring and accessing diverse data sources, such as electronic health records, imaging modalities, wearable devices, and genetic databases, and integrating this data into a coherent digital twin model can be challenging^79,80^. Health data is typically stored in a variety of formats and systems. It is difficult to overcome interoperability issues among different data formats and medical coding systems, where systems can seamlessly exchange and use data, especially challenging to incorporate real-time data which requires constant data streaming and synchronization. There is a lack of standards and interoperability for constructing DTs for health. Thus, standardized data formats and interoperability standards need to be established.

#### Data privacy and security

DTs for health rely on extensive health data of a human being, including sensitive personal health information. Ensuring privacy and security is paramount. Access to the necessary health data while protecting patient confidentiality can be a significant challenge. Another challenge of DTs for healthcare is the data privacy and security. DTs rely on the collection and analysis of extensive patient data, including sensitive health information. Safeguarding this data against unauthorized access, breaches, or misuse is a critical challenge. Compliance with regulations, such as HIPAA (Health Insurance Portability and Accountability Act) and GDPR (General Data Protection Regulation), adds complexity. Strict measures must be in place to ensure patient privacy, data encryption, secure storage, and compliance with relevant data protection regulations, both at rest and in transit, to protect it from unauthorized access.

#### Data quality and accuracy

The accuracy and quality of health data are critical. Inaccurate or incomplete data can lead to incorrect digital twin representations and, consequently, unreliable insights. However, access to comprehensive and high-quality health data is often constrained. Data may be fragmented across various healthcare institutions, making it difficult to collect a comprehensive dataset. In addition, health data is often noisy and can contain biases due to various factors, including sensor inaccuracies, patient variation, or data entry errors. Furthermore, Creating and maintaining DTs for health that evolve over time to represent changes in a person’s health or a biological system requires access to longitudinal data. This data is often scarce and may have gaps. gaps or missing data can hinder the creation of an accurate digital twin, especially in healthcare settings where not all patient data may be available. In addition, it is labor-intensive and subject to human error to generate properly labeling data, particularly in medical imaging or other diagnostic applications. Thus, maintaining data quality over time and across different sources can be challenging.

#### Data bias and fairness

Data bias poses another challenge. Data quality, completeness, and representativeness are central for the quality of DTs. The accuracy of DTs requires a data model built on a balanced dataset where any individual’s data can be compared. However, health data can be biased in various ways, such as being skewed towards certain demographics or conditions (e.g., racial, gender, or other demographic sources of bias). Building human DTs by using biased datasets would exacerbate the existing bias and eventually produce a suboptimal recommendation system, which can cause inequalities in health care^81^. Ensuring that the digital twin models are free from biases and that they do not discriminate against individuals or groups is vital.

#### Ethical considerations

Building digital twins for health presents a host of ethical considerations. Ethical considerations include but are not limited to obtaining informed consent from individuals for data collection and usage in digital twin development, addressing data ownership and control, providing patient autonomy, and identifying legal constraints. Additionally, healthcare equity must be maintained, ensuring that DTs for health do not exacerbate existing health disparities. Ethical guidelines for responsible and secure data sharing, data anonymization, and informed consent should be implemented in developing health-related DTs to foster trust and ethical practices. In addition, ensuring data accuracy and preventing biases in models is vital to avoid adverse effects on individuals, as mentioned above.

#### Modeling

Modeling also poses a challenge due to the complexities of human behaviors in real-world environments and human body structures involving a vast number of dynamic impacting factors, and sophisticated causal relations. Various socioethical considerations must be addressed in the DT healthcare space before an acceptable DT4H can be deployed. While DTs have the potential to deliver significant societal benefits and function as a social equalizer, they can also be a driver for inequality as patterns identified across populations may lead to segmentation such that the DT technology may not be accessible to everyone^82^. Privacy and high costs of DT healthcare system development are some of the main considerations as these may lead to inequities and injustice and widening of socioeconomic gap and introducing an additional instance of digital divide^83–85^. It is expected that future work in this area will focus on multi-scale models capturing measurements about the target phenomenon (disease, anatomical systems, etc.) across different scales of observation.

#### Computing infrastructure

Future advancements in high performance computing could provide the processing power necessary for more complex and accurate DT modeling and simulations. As more advances are made in Big Data and Analytics and IoT devices and sensors, the detailed or high fidelity biometric and environmental data could be used to make digital twins more accurate and useful. 5 G broadband cellular technology (and beyond) will afford faster data transfer rates and lower latency which would enable real-time updates to a digital twin, leading to further enhancements in accuracy. Conceptually, AR/VR (augmented reality/virtual reality) technologies could be used by health care researchers and providers to interact with digital twins in a more immersive and intuitive way. Finally, with blockchain and DLT (Distributed Ledger Technology), data could be stored and transferred in a decentralized, secure, and transparent manner, resulting in enhanced data privacy.

#### Business models

Finally, in order for digital twin health platforms to grow beyond academic research, compelling business models will also need to be developed that will create a market for digital twins in the health industry. Although personalized digital twin models for consumer behavior have been successful, the widespread adoption of digital twin models in medicine may take some time. Similar to the early use of digital twin simulations by NASA, it is likely that government and military organizations might be the early adopters of digital twin health systems – not only for astronauts, but also to monitor modern soldiers on special missions.

### Recommendations

Addressing these challenges requires a multidisciplinary approach involving collaboration between healthcare professionals, data scientists, technologists, policymakers, patients, and patient advocates to establish robust frameworks, standards, and guidelines for the development, implementation, and utilization of digital twins in healthcare.

First, governance structures must be in place to safeguard the rights of individuals that have DTs, support data security and privacy, and foster both transparency and fairness in the usage of data at a societal level^12^. Compliance with regulatory frameworks, ethical guidelines, and ensuring transparency and accountability are critical for the wide applications of DTs in healthcare.

Second, healthcare systems often have diverse data sources and formats, making it challenging to integrate and synchronize data from multiple sources into a cohesive DT. Data interoperability standards and protocols must be established to enable seamless data exchange and integration across different healthcare systems, devices, and platforms.

Another recommendation is to set up the infrastructure for DT implementation, which requires significant computational resources and infrastructure to handle the increasing volume, velocity, and complexity of data. Healthcare organizations need to invest in scalable and reliable computing infrastructure to support the growing demands of DT applications.

## Conclusions

DT4H is an emerging technology trend that has gained momentum in recent years, particularly during the COVID-19 pandemic. It has the potential to revolutionize healthcare by integrating with the healthcare sector, IT and AI industries, and the government. DT4H offers tremendous opportunities for personalized healthcare, predictive interventions, remote monitoring, and medical research advancements. However, it also poses challenges in terms of technological innovation, ethical considerations, societal impact, and legal guidance. Collaboration among stakeholders is crucial to harness the full potential of DT4H and ensure responsible and ethical deployment in healthcare. With interdisciplinary efforts, DT4H has the potential to transform healthcare delivery and contribute to a healthier and more connected human world.
